# Human mesenchymal stem cell differentiation on bioactive glass-coated implants

**DOI:** 10.6026/973206300222533

**Published:** 2026-04-30

**Authors:** B.S. Vinay Dutta, Omkar Shete, Anuradha Venkataramani, Anuradha Nemane, Pankaj Prakash Kharade, Priyanka T.S

**Affiliations:** 1 Department of Prosthodontics, DA Pandu Memorial RV Dental College, Bengaluru, India; 2 Department of Prosthodontics, GD Pol Dental College and Hospital PG Institute, Kharghar, Navi Mumbai, India; 3 Department of Prosthodontics, Dr. ZA Dental College, AMU Aligarh, India

**Keywords:** Bioactive glass (BG), mesenchymal stem cells, osteogenic differentiation, titanium implants, surface coating, osseointegration

## Abstract

Successful osseointegration of titanium implants remains a major challenge because the ideal bioactive surface coating and its specific influence on stem cell osteogenic differentiation are not yet fully understood. Therefore, it is of interest to evaluate the effect of bioactive glass (BG) coatings on the osteogenic differentiation of human bone marrow mesenchymal stem cells cultured on titanium implant discs. Forty-eight commercially pure titanium discs were divided into four groups (n = 12 each): uncoated control, 45S5 BG-coated, strontium-substituted BG-coated (Sr-BG) and zinc-substituted BG-coated (Zn-BG) and assessed at days 7, 14 and 21 for cell viability, alkaline phosphatase activity, osteocalcin expression, mineralized nodule formation and expression of RUNX2, COL1A1, OCN and OPN. All BG- coated groups demonstrated significantly greater cell viability, ALP activity and mineralized calcium deposition than the uncoated control (p < 0.05), while the Sr-BG group showed the highest RUNX2 and osteocalcin gene expression at day 21 (p < 0.001), followed by the Zn- BG group. Bioactive glass coating, particularly strontium-substituted BG, significantly enhanced osteogenic differentiation of hBMSCs on titanium surfaces and may improve the clinical success of osseointegration.

## Background:

The quality and speed of osseointegration at the bone implant interface is the key determinant of the long-term clinical success of endosseous implants in dental and orthopedic applications. Titanium and its alloys, which are commercially pure, are still the gold standard in the fabrication of implants because of their high biocompatibility, corrosion resistance and good mechanical characteristics [1]. Titanium is, however, intrinsically bioinert, i.e., does not actively induce bone formation, or induce differentiation of progenitor cells into osteogenic lineage cells [2]. This restriction has a clinical consequence in cases where the disease affects the quality of bone, including in the case of patients with osteoporosis, diabetes, or after irradiation therapy, where delayed or no osseointegration is a well-established problem [3]. The current interest has thus shifted to surface modification strategies to convert passive surfaces of implants into bioactive platforms that will enhance faster peri-implant bone healing. Out of all the above researched modes, bioactive Glass (BG) coatings have received and continue to receive much interest due to the distinct ability to connect hard and soft tissues by developing a hydroxycarbonate apatite layer when exposed to physiological fluids [4]. The comprehensive study since the development of the original composition 45S5 Bioglass established that bioactive glasses induce osteoblast growth, angiogenesis and have angiogenic properties due to the controlled release of ions [5]. The biological process of active biomolecule of these glasses is the dissolution and release of important ions, such as silicon, calcium, sodium and phosphorus, which react with the surrounding biological milieu to increase the expression of osteogenic genes and promote growth factors formation [6]. Recent research studies have also examined compositional alterations of the original 45S5 formulation by inclusion of therapeutic ions like strontium, zinc, copper and silver to confer more biological capabilities [7]. Of particular interest has been strontium due to its dual effect on bone metabolism, which facilitates bone formation through the action of osteoblasts and prevents bone resorption through the action of osteoclasts [8]. As an essential trace element that is involved in over 300 enzymatic reactions, zinc is an important component in bone homeostasis that triggers the differentiation and mineralization of osteoblasts and inhibits the differentiation of osteoclasts [9]. Human mesenchymal stem cells are a perfect cell model in assessing the osteoinductive ability of biomaterial surfaces since the cells are multipotential progenitor cells which could differentiate into osteoblasts, chondrocytes and adipocytes in the presence of the right stimuli [10]. The contact between the surface properties of implants and stem cell functions regulates the early cellular reaction that eventually defines the quality of the osseointegration [11].

A number of studies have also shown that bioactive glass particles and scaffolds can cause mesenchymal stem cell differentiation into the osteogenic lineage in three-dimensional culture systems [12]. Nevertheless, there are fewer studies that have conducted a systematic comparison of the impact of ion-substituted bioactive glass-based coating placed directly onto titanium implant surfaces on more extensive osteogenic differentiation parameters, including those based on proteins and those based on the genes measured longitudinally through a complete differentiation cycle. Moreover and although the biocompatibility of individual bioactive glass compositions has been establish before, a direct comparative evaluation of standard 45S5, strontium-substituted and zinc- substituted bioactive. Glass coatings on the identical titanium substrate using the same culture medium and an extensive suite of osteogenic markers have not been done in the literature yet [13]. Therefore, it is of interest to evaluate the effects of uncoated titanium, 45S5 bioactive glass-coated titanium, strontium-substituted bioactive glass-coated titanium and zinc-substituted bioactive glass-coated titanium surfaces on the viability, proliferation and osteogenic differentiation of human bone marrow-derived mesenchymal stem cells over a 21-day culture period.

## Materials and Methods:

## Study design:

This is an *in vitro* experimental study that was controlled and performed at the Biomaterials and Stem Cell Research Laboratory in the month of January to September 2024.

## Sample preparation:

Titanium alloy was cut into 48 discs (15 mm diameter x 2 mm thick) by machining the titanium rods and then the discs were successively polished using silicon carbide paper with 400-, 800- and 1200-grits. Ultrasonic cleaning in acetone, ethanol and deionized water was done in the following manner: 15 minutes each and the discs were dried under the laminar flow of air.

## Synthesis and coating of bioactive glass:

The sol-gel method was used to prepare three bioactive Glass compositions:

[1] 45S5 BG: 45% SiO_2_, 24.5% Na_2_O, 24.5% CaO, 6% P_2_O_5_ (mol %)

[2] Sr-BG 45 percent of Silica, 24.5 percent of Na2O, 18.5 percent of CaO, 6 percent of SrO, 6 percent of P2O5 (mol percent), with strontium partially substituting calcium.

[3] Zn-BG: 45 percent SiO2, 24.5 percent Na2O, 20.5 percent CaO, 4 percent ZnO, 6 percent P2O5 (mole percent), zinc partly replacing calcium.

The precursors were tetraethyl orthosilicate, triethyl phosphate, calcium nitrate tetrahydrate, sodium nitrate, and strontium nitrate and zinc nitrate hexahydrate. The sols were allowed to age at room temperature during 72h and the resulting gels were dried at 120°C during 24 hours and heat-treated at 600°C during 2 hours to stabilize the glass network. The titanium discs were dip-coated (withdrawal rate: 10 cm/min, three dipping cycles) and then sintered at 800°C during 1 hour in an argon atmosphere. There were twelve discs left uncoated as controls. There were therefore 12 discs per group (n = 12).

## Surface characterization:

FE-SEM (JEOL JSM-7600F) was used to analyse coating morphology at an electron beam of 15 kV. The composition at the element level was confirmed using energy-dispersive X-ray spectroscopy (EDS). Roughness on the surface was determined with the help of non-contact optical profilometer (Bruker ContourGT-K). The phase composition was verified using X-ray diffraction (XRD; Bruker D8 Advance). The thickness of the coatings was determined on the cross-sectional FE-SEM images.

## Cell culture:

Cryopreserved human bone marrow derived mesenchymal stem cells (hBMSCs; Lonza, passage 3-5) were cultured in a growth medium of Dulbecco modified Eagles media (DMEM) supplemented with 10% of fetal bovine serum, 1% penicillinstreptomycin and 2mm of L-glutamine at 37°C in a humidified environment of 5% CO2 atmosphere. Flow cytometry was used to characterize mesenchymal stem cell markers (CD73+, CD90+, CD105+, CD34- and CD45-), before the cell could be seeded. The purpose of 24-well plates was to put the sterilized titanium discs (autoclaved at 121°C 20 min, then UV exposure 30 min). Cells were planted with 2 x 104 cells per disc and left to settle after which 1 mL culture medium was added after 4 hours. Osteogenic induction medium (dextamethasone, 10 nM, ascorbic acid, 50ug/mL, 24 hours after seeding and 8 changes every 48 hours) was applied to differentiation assays.

## Inclusion and exclusion criteria:

The criteria used in the inclusion were the use of passages 3 to 5 of hBMSCs, which met the typical mesenchymal stem cell surface marker profile, titanium discs and no visible surface defects and uniform coatings as determined by FE-SEM. This was on exclusion criteria of discs that showed coating delamination, microbial contamination of cell cultures at any time and cell viability below 80% at initial seeding evaluation. The assay of cell viability and proliferation was performed using a resinogenic solution consisting of the following components: 10 mg/L NaCl, 0.2 mg/L NaOH, 0.04g/L Na3, 0.04g/L NaOCl, 0.88g/L NaH2O, 10 ml/L water, 0.05ml/L resinogenic solution. The Cell Counting Kit-8 (CCK-8; Dojindo Laboratories) colorimetric assay was used to evaluate cell viability and proliferation on days 1, 3, 7, 14 and 21. The absorbance at 450 nm was determined with a microplate reader (BioTek Synergy H1). The results were given in optical density (OD) values. Alkaline phosphatase activity was determined using an ELISA kit. ELISA kit was used to determine Alkaline phosphatase activity. Measures of ALP were taken at 7 and 14 days. Triton X-100 fixed cells were lysed and the ALP activity was measured with a p-nitrophenyl phosphate substrate kit (Sigma-Aldrich). The activity of the enzyme was adjusted to total protein content according to the bicinchoninic acid (BCA) assay and converted to nmol p-nitrophenol/min/ mg protein.

## Mineralization assay:

At day 21, alizarin red S staining was done to determine calcium deposition. Cells were fixed in 4% paraformaldehyde, stained with 40 mM of Alizarin red S (pH 4.2) after 20 minutes and rinsed thoroughly. Semi-quantitative analysis was done using bound stain that was eluted with 10 per cent cetylpyridinium chloride and measured at 562 nm. Using a qRT-PCR, a real-time PCR can be conducted in the presence of amplification dyes. A real-time PCR with amplification dyes can be performed using a qRT-PCR. At days 7, 14 and 21, a total RNA was isolated using TRIzol reagent (Invitrogen). The synthesis of complementary DNA was done on a High-Capacity cDNA Reverse Transcription Kit (Applied Biosystems) and the qRT-PCR was conducted on an Applied Biosystems QuantStudio 5 system with SYBR Green Master Mix. These were target genes such as RUNX2 (runt-related transcription factor 2), COL1A1 (collagen type I alpha 1), OCN (osteocalcin/BGLAP) and OPN (osteopontin/SPP1). GAPDH was the housekeeping gene.

## Statistical analysis:

Each disc was subjected to all experiments in triplication and the data was presented as mean standard deviation. The statistical analysis was done with the SPSS software (version 28.0; IBM Corp., Armonk, NY). The Shapiro-Wilk test was used to test normality. Analytical differences between groups at a given time were conducted through one-way analysis of variance (ANOVA) and then using Tukey honestly significant difference (HSD) post-hoc test to contrast the difference between the groups. ANOVA was employed in analyzing the interaction between group and time through two-way ANOVA. The level of statistical significance was defined as p < 0.05.

## Results:

Alkaline phosphatase (ALP) activity and Alizarin Red S quantification demonstrated statistically significant differences among the study groups, as presented in Table 1 (see PDF). At day 7, ALP activity was significantly higher in all bioactive glass (BG)-coated groups compared to the control group (p < 0.001), with the Sr-BG group exhibiting the highest values (24.3 ± 3.1), followed by Zn-BG (21.1 ± 2.5) and 45S5 BG (19.6 ± 2.8). At day 14, a further increase in ALP activity was observed across all groups, with the Sr-BG group again demonstrating the highest activity (45.8 ± 5.3), which was significantly greater than both 45S5 BG and Zn-BG groups (p < 0.001). The Zn-BG group (38.2 ± 4.0) also showed significantly higher values than the 45S5 BG group (32.5 ± 4.2), while all BG-coated groups remained significantly elevated compared to the control (18.7 ± 3.0). Alizarin Red S quantification at day 21 revealed a similar trend, indicating enhanced mineralization in BG-coated groups. The Sr-BG group exhibited the highest mineral deposition (1.58 ± 0.18), followed by Zn-BG (1.21 ± 0.15) and 45S5 BG (0.91 ± 0.12), all of which were significantly greater than the control group (0.42 ± 0.08) (p < 0.001). Post hoc analysis confirmed that all BG-coated groups showed statistically significant improvements compared to the control group, while the Sr-BG group consistently demonstrated superior performance compared to both 45S5 BG and Zn-BG groups. These findings indicate that strontium incorporation enhances osteogenic activity and mineralization potential more effectively than conventional and zinc-modified bioactive glass coatings. Osteogenic gene expression data at days 7, 14 and 21 are presented in Tables 2 and 3 (see PDF). Two-way ANOVA revealed significant main effects of both group (p < 0.001) and time (p < 0.001) as well as a significant group x time interaction (p < 0.001) for all measured parameters, indicating that the magnitude of the coating effect increased progressively with culture duration.

## Discussion:

The current paper has conducted a systematized assessment of the osteogenic capacity of three bioactive glass coating formulations placed on titanium implant surfaces in terms of a complete set of differentiation markers in a 21-day culture timeframe. The null hypothesis was disproved as the experimental groups showed significant differences in cell viability, ALP activity, mineralization and osteogenic gene expression with the strontium-substituted bioactive glass coating showing the strongest pro-osteogenic activity. The increased cell proliferation upon all the bioactive glass-coated surfaces relative to the uncoated titanium can be linked to the long- established bioactive behavior of silicate glasses, which dissolve their surface in order to release the ionic species that induce the cellular metabolic activity [14]. The hydroxycarbonate apatite deposition onto bioactive glass surfaces offers a biomimetic surface on which cells become attached via integrin-mediated signaling as has already been demonstrated in research involving the study of surface-mediated cell responses [15]. Also, the roughness of coated discs as inspected by profilometry in the current paper is likely to have assisted in better initial cell adhesion and eventual proliferation due to higher surface area and positive topographical signals [16]. The fact that the ALP activity was higher, significantly at days 7 and 14 across all coated groups and especially in the Sr-BG group, implies an early commitment towards osteogenesis. ALP is a well-known early indication of osteoblastic differentiation and a preconditioning enzyme of mineralizing the matrix [17]. The gradual rise in ALP activity between day 7 and day 14 followed by plateau or low-grade decrease between day 14 and day 21 that was observed through the coated groups is typical of regular osteogenic maturation and has been noted in many stem cell differentiation studies [18]. The most notable result of this study was the considerably high performance of the strontium substituted bioactive glass coating in all most of the parameters considered. Sr-BG group showed RUNX2, COL1A1 and OCN expression upregulation of 4.2-fold, 6.3-fold and 6.9-fold at day 21 as compared to the control. These findings are consistent with the growing body of evidence indicating that strontium ions strongly stimulate osteoanabolic activities by activating the calcium-sensing receptor and downstream signaling pathways, which include the Wnt/ -catenin, Ras/MAPK and NFATc pathways [19]. Activation of calcium-sensing receptor by strontium has been demonstrated to induce nuclear translocation of β-catenin that in turn interact with the T- cell factor/lymphoid enhancer factor transcription factors to induce the expression of osteogenic genes such as RUNX2 which is the master transcriptional regulator of osteoblast differentiation [20]. Moreover, it is likely that the continuous release of strontium out of the glass matrix during the 21-day culture period was a continuous stimulus on the osteogenic pathways which is evident by the progressive and increasing differences between the Sr-BG and other groups at late points in time. The ability to release ions in a controlled manner is also a major benefit of glass-based delivery systems to direct supplementation of culture medium with strontium salts because this behavior also replicates the time-dependent release characteristics that would otherwise take place *in vivo* at the bone-implant interface [21]. The zinc-substituted bioactive glass coating also showed much better osteogenic differentiation than the uncoated control and 45S5 BG group, although its influences were always minor compared to those of the Sr-BG formulation. Zinc stimulates osteoblast differentiation by several pathways, such as the stimulation of the activity of ALP, the growth of collagen and the direct activation of aminoacyl-tRNA synthetase complex that is the factor of the protein translation [22]. Also, zinc was demonstrated to suppress osteoclast development by blocking the RANKL/RANK signaling axis which, although not directly evaluated in the current mono-culture model, would add some extra benefits *in vivo* [23]. Of specific interest should be the strong upregulation of late-stage osteogenic markers especially the osteocalcin and osteopontin in the Sr-BG and Zn-BG groups in day 21. Osteocalcin is the most concentrated non-collagenous bone matrix protein and is very important in the maturation of mineral crystals, as well as calcium homeostasis and osteopontin is a bridging molecule between the cells and the mineralized matrix [24]. The correlation of these markers with the quantitative mineralization measurement of the Alizarin red staining data is a good indication that the ion-modified bioactive Glass surfaces stimulate the initiation as well as the maturation and completion of the osteogenic differentiation program. The excellence of the standard 45S5 BG coating over the uncoated controls confirms the inherent osteoinductive potential of traditional bioactive glass compositions which has clearly been established in the literature [25]. Silicon and calcium ions that escape during the breakdown of 45S5 glass independently stimulate osteogenesis by increasing the production of collagen types I and raising the secretion of IGF-II, respectively [26]. Nevertheless, the much more pronounced effects on the case of ion-substituted formulations suggest that compositional tailoring can have a considerable enhancement of the intrinsic bioactivity of the base glass. The sol-gel dip-coating technique used in this experiment generated monolithic, uniform and adhesive layers of suitable thickness to be used in clinical translation. Past studies have shown that coating thicknesses between 5 and 15 μm give an optimum balance between adequate ion reservoir space to support long term biological functions and mechanical stability of the coating under working load conditions [27]. The amorphous property of the coating, as revealed by XRD is likely to enhance quicker dissolution rates than crystalline glass-ceramics which could be beneficial in hastening early-stage osseointegration [28]. There are a number of constraints of this research that could be mentioned. To begin with, the monoculture system with hBMSCs fails to recapitulate the complicated multicellular environment at the bone implant interface *in vivo* where osteoclasts, endothelial cells, immune cells and fibroblasts interact to coordinate bone remodeling. Second, the mechanical loading conditions, which implants undergo during clinical functioning, were not modelled and the stability of the coatings in cyclic loading ought to be investigated separately. Third, although the 21-day culture period is common in *in vitro* studies of osteogenic differentiation, this period does not reflect long-term remodelling events that take place in clinical osseointegration. Fourth, a single concentration of the replenished ion was assessed and dose-response correlations ought to be researched in subsequent studies. Lastly, the antibacterial efficacies of the coating material, especially when zinc is used, were also not evaluated and constitute a noteworthy direction to further research [29]. Nevertheless, this study has several limitations but the resultant data is important in providing comparative data on osteogenic performance of ion-substituted bioactive glass coatings and give a rational foundation to prioritize strontium-substituted formulations in preclinical animal studies to assess the role of bioactive glass in the future in evaluating the osteogenic performance. The scale of the difference in the level of expression observed, especially the almost seven-fold upregulation of the osteocalcin expression in the Sr-BG group compared to the uncoated controls, indicates clinically significant possibilities of enhancing the implantation outcome of patients with impaired bone healing potential [30].

## Conclusion:

Bioactive glass coatings on commercially pure titanium significantly enhanced the osteogenic differentiation of human bone marrow- derived mesenchymal stem cells compared with uncoated titanium surfaces. Among the tested formulations, strontium-substituted bioactive glass demonstrated the strongest pro-osteogenic effect, while zinc-substituted bioactive glass also performed better than standard 45S5 bioactive glass and uncoated control surfaces. These findings support the potential of ion-substituted bioactive glass coatings, particularly strontium-based coatings, for improving implant bioactivity, although further *in vivo* and long-term studies are required before clinical application.
